# Cherokee Medical Association

**Published:** 1890-03

**Authors:** J. F. Johnson, J. H. Evans

**Affiliations:** Vice President; Rusk; Secretary; Rusk


### CHEROKEE MEDICAL ASSOCIATION.

The Cherokee County Medical Association held its regular meeting on the 2nd inst. The President being absent, Dr. J. F. Johnson, Vice-President, called the meeting to order.

The meeting was pleasant and profitable. The ordinary business being finished, the reading of papers was called. Dr. B. F. Brittain, of Jacksonville, responded with an interesting paper on the importance of "Proper Diagnosis in Female Troubles." The paper was well received. Dr. Brittain is an old practitioner, posted in his profession and his advice on those important subjects may well be listened to.

The paper elicited remarks from several of the members, and by unanimous vote was directed to be sent to DANIEL'S TEXAS MEDICAL JOURNAL for publication.

The Committee on Collective Investigation then reported through Dr. L. D. Guinn on "Black Jaundice, its Etiology, Pathology, Treatment, etc."

The etiology was generally believed to be malarial poison. The pathology, from the continued effects of the poison upon the system, the liver fails to act properly and in consequence thereof the bile is eliminated through the skin and kidneys, thus producing the yellow appearance of the skin, and, by breaking down the small blood vessels, hemorrhage of the kidneys. The treatment provoked most discussion. Dr. B. F. Brittain gives calomel in small doses first, last and all the time. He does not use any special diuretics, nor does he object to quinine, but thinks there are cases in which it may be used to advantage, and would give morphine and atropine, if necessary. Dr. Frazer said, he used calomel and gave diuretics for all they are worth. He gives ergot, cannabis indica, etc., to control hemorrhage in some cases, and does not think he ever saw a case die from loss of blood, but thinks they usually die from suppression of urine; uses morphia and atropia to control vomiting and produce rest. Counter irritation to stomach and spine, quinine when indicated. Dr. Evans uses calomel and diuretics, quinine towards the last and does not think opiates admissible, and when given it is done at the expense of the kidneys; uses counter irritation. Drs. Guinn, of Rusk, and Smith, of Gent, both use about the same treatment as the rest, except they differ from Dr. Evans in the use of opiates. Dr. Smith uses opium and laudanum, Dr. Guinn morphia and atropia hypodermically.

All agree that in this formidable enemy elimination, generally, is of great importance. After this lively discussion the Association elected its officers for the ensuing year: Dr. B. F. Brittain,

President; Dr. A. H. McCord; First Vice President; Dr. J. H. Evans, Second Vice President; Dr. J. R. Roland, Third Vice President; Dr. J. K. Frazer, Secretary; Dr. W. B. Pullen, Assistant Secretary.

Drs. McCord, Evans, and Guinn, of Rusk, constitute the Committee on Collective Investigation.

The essayists and subjects are as follows: Dr. McCord, Post Partem Hemorrhage. Dr. Collins, The Best and Safest Anæsthetic. Dr. J. N. B. Guinn, Slow Fever. Dr. Roland, Physiological Action of Atropine.

The next meeting will be held at Alto, on the first Tuesday in April. J. F. JOHNSON, M. D., Vice President.

J. H. EVANS, M. D., Secretary.

RUSK, January, 1890.

